# Evaluation of In-Situ Low-Cost Sensor Network in a Tropical Valley, Colombia

**DOI:** 10.3390/s25041236

**Published:** 2025-02-18

**Authors:** Laura Rojas González, Elena Montilla-Rosero

**Affiliations:** 1 Eafit University, Medellín 050022, Colombia; lrojasg3@eafit.edu.co; 2 Applied Sciences and Engineering, Natural Systems and Sustainability Department, SOPHIA Research Group, Eafit University, Medellín 050022, Colombia

**Keywords:** low-cost sensors, sensor validation, urban air quality monitoring, particulate matter 2.5

## Abstract

The increase in yearly particulate matter concentrations has been a constant issue since 2017 in the Aburrá Valley, located in Antioquia, Colombia. Although local certified air quality monitors provide high accuracy, they are limited in spatial coverage, limiting chemical transport and pollution dynamic studies in this mountainous environment. In this work, a local, Low-Cost Sensor network is proposed as an alternative and has been installed around the valley in representative locations and heights. To calibrate PM_2.5_ and O_3_ sensors used by the network, temporal delays were analyzed with Dynamic Time Warping and the linear scale was corrected with a Single Linear Regression model. As a result, the correlation coefficient R^2^ of the sensor reached values of 0.8 and 0.9 after calibration. For all network stations, rescaled data agrees with official historical reports on the behavior of pollutant concentrations and meteorological variables. The ability to compare the network results with certified data confirms the success of the calibration/validation method employed and contributes to the growing field of low-cost air quality sensors in Latin America.

## 1. Introduction

Air quality is one of the most significant environmental causes related to human health and biodiversity preservation; pollutants like particulate matter 2.5 (PM_2.5_) and ozone (O_3_) aggravate human illnesses and climate change [1,2]. Growing urban areas have the most diverse human activities; industrial processes and vehicular traffic mainly cause higher concentrations of pollutants [3,4]. However, due to the rapid increase in public awareness of air pollution, more urban air quality monitoring has been requested in recent decades. Local governments work to expand and enhance the networks of air quality monitors. Even so, according to the WHO [5], more than 90% of air quality monitoring facilities around the globe are mainly in China, Europe, India, and North America.

Standard air quality monitoring yields accurate and high-quality data; it is costly, requires specialized operators, and is not feasible for widespread citizen coverage because of its fixed location. The insufficient spatial representation impairs the study of the dynamics of gas and pollutant particles, creating a need for alternative measuring methods [2]. This pattern is changing with the availability of low-cost, easy-to-use air pollutant sensors that can provide real-time measurements at an affordable price [6]. While high-end sensors can cost tens of thousands of US dollars and even millions annually for maintenance, low-cost sensors (LCS) can cost up to 500 USD for optical sensors, with much lower-priced alternatives like metal oxide sensors for 15 USD.

Furthermore, LCS can provide real-time measurements thanks to operating principles, and many of them are designed to send measurement data by internet networks (e.g., WiFi, radiofrequency) [2,6,7,8,9]. Other properties depend on the type of sensor, i.e., electrochemical sensors based on the chemical reaction produced by the atmospheric gases. They are generally employed to measure Nitrogen Oxides (NO_2_), Sulfur Dioxide (SO_2_), Carbon Monoxide (CO) and O_3_, with a high sensitivity to temperature or humidity changes [6,8,10,11,12,13]. In metal oxide sensors (MOS) the surface resistance is modified by the gases in the air. This type of sensor commonly measures gaseous Hydrogen, Carbon Oxides, Nitrogen Oxides and Methane (H_2_, CO_x_, NO_x_, SO_x_, and CH_4_, respectively) [12,13,14,15]. Photoionization detectors ionize volatile organic compounds (VOC) to measure the resulting electrical current. They have a limited dependency on temperature and humidity and a significant signal drift [16,17]. Optical particle counters detect particulate matter by measuring scattered light for later theoretical calculation of particle size depending on shape, color, density, humidity, and refractive index. These are different from optical sensors, which detect gases such as CO and Carbon Dioxide (CO_2_), by measuring the absorption of infrared light [6,8,12,16,17].

LCS networks can complement traditional monitoring methods, increasing spatial coverage and improving exposure estimates due to their greater accessibility [11,12,18,19,20]. However, the quality of LCS data remains a significant concern, as their performance can be affected by atmospheric conditions and cross-sensitivities [6,16,17]. This has contributed to the lack of a standard protocol for calibration procedures to increase data accuracy [13,21]. As a result, long-term applications (i.e., periods longer than 3 months) of low-cost air quality networks remain a challenge [22]. Another significant issue is the unequal access to global LCS data: about 41% represents North America, 24% represents Asia, and less than 15% represents South America and Africa combined [23,24], restricting the understanding of air quality in regions that would benefit from low-cost alternatives. Despite limitations, LCS technology is promising for qualitative analysis in indoor and outdoor environments, encouraging its use while acknowledging the continued importance of high-quality instruments [25,26,27,28,29].

In this study, we evaluated the reliability of air quality measurements taken by innovative, low-cost devices deployed in a tropical valley. Our main contribution is a comprehensive long-term field assessment of a low-cost PM_2.5_ and trace gas detector tailored for tropical environments. These environments are characterized by consistently high temperatures and humidity, and a variety of urban and semi-urban locations situated between 1400 and 2160 m above sea level. Consequently, this evaluation provides significant insights into the performance of LCS in challenging environmental conditions within an under-sampled geographic region.

This work has been organized according to the following reasoning. The technical details of the sensors, the measurement site and the network description planned for data collection are presented in Section 2. The methods developed for pre-processing and processing data are described in the same section. Section 3 presents the results of the validation and calibration process applied to a field campaign measurement, performed by colocating it with reference instruments. The measurements of the LCS network over a year, along with a preliminary analysis of the network measurements considering annual, monthly, and daily average values of PM_2.5_ and O_3_, are presented in Section 3. Finally, Section 4 presents the summary of the findings of this investigation along with potential future uses.

## 2. Methodology

### 2.1. Low-Cost Sensor Device

**Figure 1:**
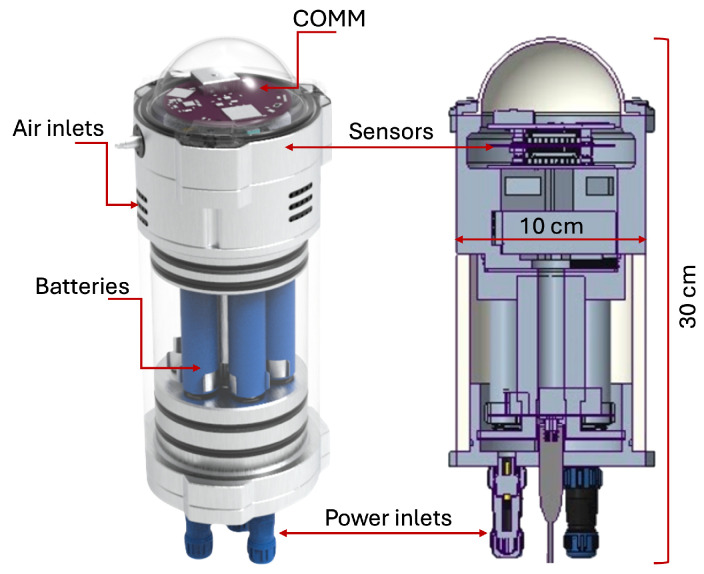
Simple LCS unit and cross section, labeled main parts.

For this study, we employed an LCS with a design-resistant, non-invasive, and modular-based CanSat development (a CanSat is a standard pico-satellite form factor soda can satellite) named Simple unit, with a cylindrical array structure. This device has evolved from several architectures [2]; the engineering design, development, and overtests that ensure the long-term performance and reliability of the devices are described in [2]. The Simple unit is shown in Figure 1, where the sensors and communication board are on top, the pin to start it on the left side, batteries in the middle, and external connecting plugs at the inferior part. It has a volume of 1.0 L and a mass of 1.5 kg.

Every Simple unit has four electronic modules: the onboard data handling subsystem manages, formats, stores, and sends data from electronic modules to the communication segments and later to SD memory. The energy power subsystem regulates the power of the battery bank with 20,000 mAh. This battery supplies between 3.0 and 4.2 V, increased to 5.0 V to power the other subsystems. It provides the option in which solar cells enable an extended operating time when installed. The communications subsystem features three types of radios (LoRa Tx/Rx @ 915 MHz, Dorji Tx/Rx @ 434 MHz, and Radiometrix Tx/Rx @ 434 MHz) and Wi-Fi connection. It ensures a minimal line of sight of 300 m without multipath propagation, rain fade, or concentrated vegetation. Finally, the payload subsystem is where the sensors are assembled. The Simple unit is equipped with sensors to measure concentrations of O_3_, NO_x_, PM_2.5_ and meteorological variables such as temperature and relative humidity. Table 1 shows the main Simple unit technical specifications; the type of sensors selected to measure each variable is the most used in global records [6].

### 2.2. Measurement Site and Network

The Aburrá Valley is a narrow valley between the western and central Andes Mountain ranges in Colombia, located in the central mountain range in Antioquia department (6.231944, −75.568056). The valley is approximately 60 km long and variable in width. Its topography is sloping and uneven, with elevations varying from 1300 to 2800 m.a.s.l. It has a total area of 1157 km^2^, and the urban area represents approximately 30% of the valley. Because of reduced ventilation and high pollution emissions from urban areas, this valley may suffer severe air pollution problems [30]. In addition, this valley has experienced air pollution from external sources like wildfires and Sahara dust [31] and is surrounded by diverse ecosystems such as Páramo landscapes and montane ecosystems [32]. Consequently, this tropical valley is a perfect natural laboratory for investigating urban pollution and its possible effects, particularly on biodiversity.

In Colombia, air pollution is a significant environmental problem that concerns all group populations due to its impacts on health and related economic costs. The policies of the country for controlling air pollution establish the maximum levels of the criteria pollutants at less stringent levels than those of the WHO and EPA. However, in the Aburrá Valley, there is the densest standard network for measuring air pollutant concentrations in the country, thanks to the Early Warning System for Medellín and the Aburrá Valley (SIATA, accessed online: https://siata.gov.co/ (accessed on 10 February 2025)). SIATA holds authority over climatic warnings in the region and serves as a reference for calibration processes, ensuring the dependability of the results. SIATA monitors real-time noise, meteorological, and hydrological variables as well as operates the Air Quality Monitoring Network using 36 manual and automatic stations across the Aburrá Valley. Most measurements are performed through the valley bottom, the heavily polluted zone. The lack of measuring stations at the higher altitudes of the mountains that surround the city limits our ability to comprehend the dynamics of pollutants in the valley and their dispersion towards surrounding ecosystems [2].

**Figure 2:**
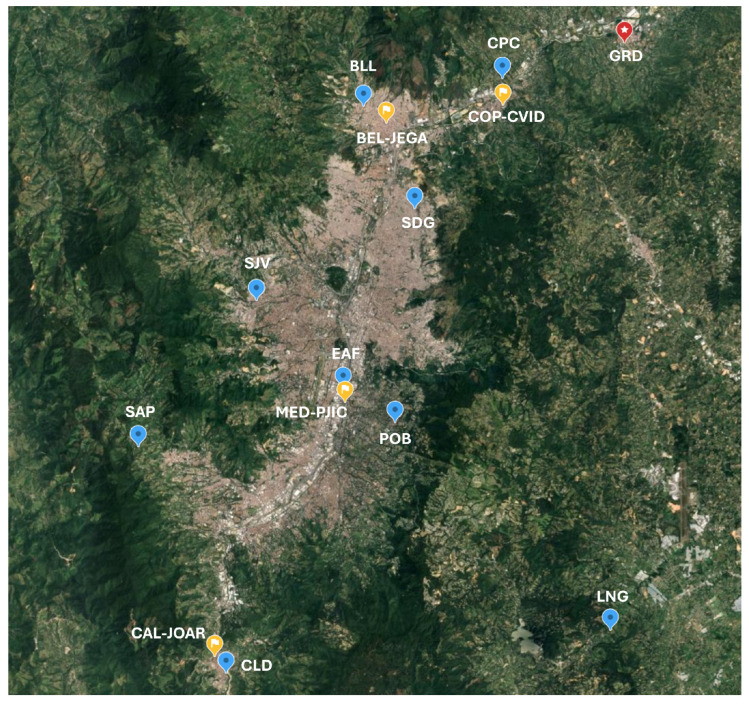
4DAir in-situ Monitoring Network in Aburrá Valley.

An in-situ network named 4DAir was installed during a local research project between August 2023 and August 2024. A star topology network was chosen to increase the spatial coverage of air quality sensors. Figure 2 shows the location of the monitoring points in blue pins, the calibration site in a red pin (GRD), and the SIATA reference stations marked in yellow pins.

Each network station has been assigned a three-letter code based on location to facilitate identification. These stations have been classified as rural (LNG and SAP), semi-urban (CPC, CLD and BLL), and urban (SDG, EAF, SJV, and POB) sectors, according to their location. The altitude difference between the lowest and highest stations is 700 m. The geographic data of the network stations are shown in Table 2.

Eight out of nine stations were installed using residential electrical supply; EAF is powered by solar cells. Only the SJV unit uses a LoRaWAN radio signal (915 MHz) to communicate with the gateway at the EAF station, while the rest use a Wi-Fi connection for data communication. Table 3 shows details such as installation date, duration, and effective days of measurements.

### 2.3. Data Evaluation

Numerous studies focusing on validation, calibration and assessment of the quality of LCS data have led the research community to embrace key performance metrics and statistical measures [6,18,20,25,33]. In this work, we used two filters for preprocessing raw LCS data. First, we removed physically unfeasible values such as zeros and negative values from measurement data. We then examined the daily time series of raw data, and using a moving average, we identified abnormal spikes relative to the neighboring data points. To increase data processing efficiency without losing physical meaning, we removed raw values outside the 95% confidence interval, defined as the mean ± 2σ, where σ is the standard deviation. This confidence interval has been used successfully in comparable long-term networks [34,35,36].

In addition, we used two correlation evaluation methods to perform calibration against the reference values. SIATA reference and Simple unit data have different temporal resolutions, 1 h and 1 min, respectively. Before applying the correlation methods, we resampled the LCS data by calculating the mean values of the last measured hour. The correlation measures the extent to which the sensor readings and reference values are related. Initially, we employed Dynamic Time Warping (DTW) to detect temporal lags between the measurements and reference time series. Subsequently, we calculated the Single Linear Regression (SLR) between raw filtered and reference values. In this study, the PM_2.5_ and O_3_ reference values were retrieved from the standard sampler (Low-Vol PM_2.5_ PQ200, BGI MesaLabs^®^, Lakewood, CO, USA) and the analyzers (Teledyne 200E and M400E, City of Industry, CA, USA) belonging to SIATA [37,38]. Reference instrumentation is calibrated according to EPA quality assurance by calibration labs in compliance with ISO/IEC 17025 [39].

Section 2.3.1 and Section 2.3.2 outline the fundamentals of the DTW and SLR methods. Finally, we used the SLR model to process data from LCS networks.

#### 2.3.1. Dynamic Time Warping (DTW) Method

DTW is a function that compares time sequences, providing an optimal alignment path and minimizing point-to-point distances. The DTW distance δ is computed as an Euclidean distance, with the exception that time samples within a sequence can be compared with multiple instant data points of the second sequence, which is known as the cost function. The optimal warping path ($min_{\pi }$) is calculated by minimizing the total cost, representing the matches with the least distance between the points. Another parameter of interest is the similarity score, the number of matches made in the best path [40,41]. This score falls between 0 and 1, where a lower number indicates greater similarity. Equation (1) defines the DTW cost function for time series *x* and $x^{\prime }$ of lengths *M* and $M^{\prime }$.

(1) $$d_{DTW}\left(x,x^{\prime }\right)=min_{\pi }\sqrt{\sum_{(j,j^{\prime })\in \pi }\delta \left(x_{j},x_{j}^{\prime }\right)={\left(x_{j}-x_{j}^{\prime }\right)}^{2}}$$

where the following boundary conditions (2) and (3) are established:

(2) $$\left(j_{1},j_{1}^{\prime }\right)=\left(1,1\right)\mathrm{and}\left(j_{P},j_{P}^{\prime }\right)=\left(M,M^{\prime }\right)$$

(3) $$\left(j_{p},j_{p}^{\prime }\right)\in \left\{\left(j_{p-1},j_{p-1}^{\prime }+1\right),\left(j_{p-1}+1,j_{p-1}^{\prime }+1\right),\left(j_{p-1}+1,j_{p-1}^{\prime }\right)\right\}$$

We are correlating datasets measured by two different kinds of methods: the optical counter in the Simple unit with the standard reference technique used by SIATA, the gravimetric method; thus, we need to take into account that they generally have different time responses, data frequency, and signal delays [16], depending on their communication protocols. Firstly, we used the DTW method to identify the signal delays in the LCS. After, due to the LCS using wireless communication, raw data might contain missing values, and the original function in Equation (1) can underestimate the similarity score because of this. Therefore, the delay presented in the Simple PM sensor is computed with the model proposed by Yurtman et al. in [40], called DTW-AROW, a DTW Python library that handles missing values without the contextual information needed. Equation (4) defines it, and boundary conditions (5)–(7) are added to the original DTW function.

(4) $$d_{DTW-AROW}\left(x,x^{\prime }\right)=min_{\pi }\sqrt{\gamma \sum_{(j,j^{\prime })\in \pi }\delta_{ext}\left(x_{j},{x^{\prime }}_{j^{\prime }}\right)}$$

where

(5) $$\delta_{ext}\left(x_{j},{x^{\prime }}_{j^{\prime }}\right)=\left\{\begin{array}{cc} 0, & \mathrm{if}\;x_{j}=\mathrm{NaN}\;\mathrm{or}\;{x^{\prime }}_{j^{\prime }}=\mathrm{NaN}\\ {\left(x_{j}-{x^{\prime }}_{j^{\prime }}\right)}^{2}, & \mathrm{otherwise} \end{array}\right.$$

(6) $$\left(j_{p},j_{p}^{\prime }\right)=\left(j_{p-1}+1,j_{p-1}^{\prime }+1\right)\;\mathrm{if}\;\mathrm{NaN}\;\in \left\{x_{j_{p}},x_{j_{p-1}},x_{j_{p}^{\prime }}^{\prime },x_{j_{p-1}^{\prime }}^{\prime }\right\}$$

(7) $$\gamma =\frac{M+M^{\prime }}{M_{av}+M_{av}^{\prime }}$$

And γ is a conversion factor that considers the number of available data (av) as well as the total lengths *M* and $M^{\prime }$ of the series.

#### 2.3.2. Single Linear Regression (SLR)

Most simplified models are linear and can be defined by Equation (8):

(8) $$Y=\beta_{0}+\beta_{1}x+\epsilon$$

In this case, *Y* represents the reference signal (SIATA information), *x* is the target signal (Simple unit data), and $\beta_{0}$ and $\beta_{1}$ are linear coefficients, intercept, and slope, respectively. Parameter ϵ usually receives the name of random error or bias, and it is a component with a constant variance. This model must include the dataset $(x_{i},y_{i});$ i={1,2,...,n}, which implies that $n=x_{i}$ pairs of (x,y). A well-selected model suggests no other regressors, and it is reasonable to include positive or negative low errors around the real model. It is, of course, a conceptual or estimated result, and the more data are available, the more accurate the model can be [42].

This model is applied to Simple unit data by defining the linear coefficients and random errors like in the expressions (9)–(11):

(9) $$\beta_{0}=\frac{std(Y)}{std(x)}$$

(10) $$\beta_{1}=mean\left(Y\right)-\beta_{0}mean\left(x\right)$$

(11) $$\epsilon =\hat{x}-\left(\beta_{0}+\beta_{1}x\right)$$

where $\hat{x}$ is the predicted signal. Note that bias is simply the difference between the reference and predicted or calculated data. Therefore, the corrected data $x_{corr}$ are estimated by Equation (12):

(12) $$x_{corr}=\beta_{0}+\beta_{1}x+\epsilon$$

### 2.4. Air Quality Index

The Air Quality Index (AQI) interprets the concentration values of each pollutant measured by an air quality monitoring device. The AQI value is a way to homogenize each pollutant’s measurements into a relevant message to users since each one has its own risks and a different concentration range where it could be in AQI categories. These categories describe the safety or hazard level each region has, considering the sensitive groups and illnesses each pollutant can cause at a certain level [43]. Equation (13) shows the AQI definition, where PM_2.5_ AQI is calculated for daily averages and O_3_ AQI is calculated for octohourly averages, following The U.S. Environmental Protection Agency’s (EPA) standard. Based on this result, each pollutant concentration is assigned a category and a color, depending on its effect on human health: Good (green), Moderate (yellow), Unhealthy for sensitive groups (orange), Unhealthy (red), Very unhealthy (purple) and Hazardous (maroon).

(13) $$I_{p}=\frac{I_{Hi}-I_{Lo}}{BP_{Hi}-BP_{Lo}}\left(C_{p}-BP_{Lo}\right)+I_{Lo}$$

where

$I_{p}$: index for pollutant *p*;
$C_{p}$: truncated concentration for pollutant *p*;
$BP_{Hi}$: concentration breakpoint that is greater than or equal to $C_{p}$;
$BP_{Lo}$: concentration breakpoint that is less than or equal to $C_{p}$;
$I_{Hi}$: AQI value corresponding to $BP_{Hi}$;
$I_{Lo}$: AQI value corresponding to $BP_{Lo}$.

## 3. Results and Discussion

### 3.1. Calibration and Validation

The performance of a Simple unit for PM_2.5_, O_3_, and NO_x_ was evaluated and calibrated by measuring co-located with a reference sampler (Low-Vol PM_2.5_ PQ200, BGI MesaLabs^®^, Lakewood, CO, USA) and reference gases analyzer (Teledyne 200E and M400E, City of Industry, CA, USA) operated by SIATA. These measurements were performed in the GRD station during a week between 15 and 22 December 2023. This field campaign aimed to assess the data quality of measurements from the LCS compared to high-quality standard equipment and develop pre-processing algorithms according to the filters presented in Section 2.3, i.e., removing zero and negative values and outliers from a confidence level of 95%. Furthermore, the correlation techniques (DTW and SLR) described in the same section were applied using these reference measurements. Because data from reference instruments are available hourly, the LCS data were converted to hourly mean values to carry out the comparison. The low-cost sensors in the network described in this work were then tested by comparing their data with the Simple unit that was co-located with reference instruments.

During co-located measurements and for the entire network of stations, we discovered that the NO_x_ sensor data were consistently an order of magnitude below reference values [37,38], despite their excellent operation range and resolution. According to [14,44], the development of gas MOS sensors is causing instabilities, and improving their output would require some external energy; these and other multiple issues are still unresolved or unknown. As a result, NO_x_ data were discarded, and the MOS MiCS-6814 is not advised for air quality monitoring if temperatures greater than 100 ºC are not reached within the LCS enclosure.

#### 3.1.1. Temporal Delay Correction

**Figure 3:**
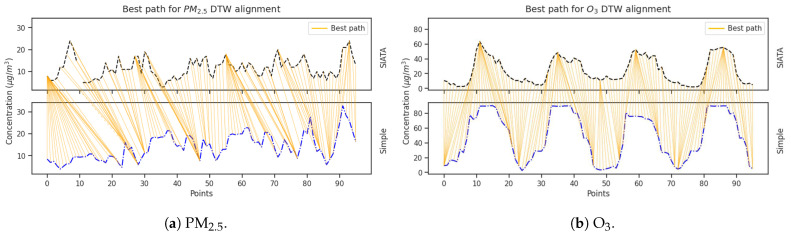
Temporal delay identification between SIATA and Simple unit signals using the DTW-AROW method. For (**a**) PM_2.5_ and (**b**) O_3_ raw signals.

**Figure 4:**
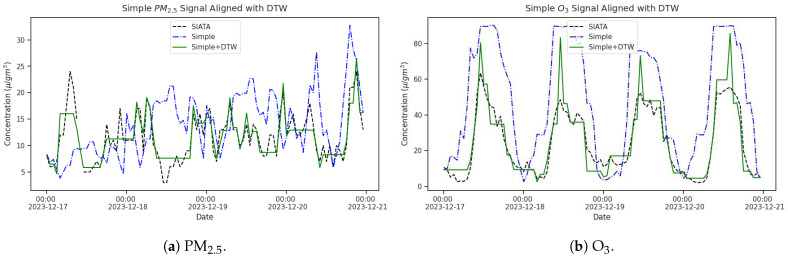
Simple unit data corrected compared with SIATA reference values and Simple unit raw signals. (**a**) PM_2.5_, (**b**) O_3_.

The DTW method working with missing values, as described in Section 2.3, was applied to PM_2.5_ and O_3_ signals considering the intercomparison field campaign information. The similarity scores retrieved for PM_2.5_ and O_3_ were 0.2 and 0.6, respectively. As Figure 3 shows, a higher temporal delay was identified for PM_2.5_ than for O_3_ measurements carried out with the Simple unit. This is noticed from the more considerable distances in the PM_2.5_ point-to-point graph (Figure 3b) than in the O_3_ one (Figure 3a), and it is consistent with the evaluation performance made for this kind of PM optical sensor [16]; meanwhile, both SIATA and Simple Unit use electrochemical sensors to measure O_3_, reducing the differences in response time. Following the recognition of the temporal delay, the Simple LCS measurements were corrected as shown in Figure 4 for both species. Once more, a more substantial correction has been noted in PM_2.5_ data as expressed in the horizontal lines in Figure 4a, demonstrating the potential of the DTW method.

#### 3.1.2. SLR Correction

**Figure 5:**
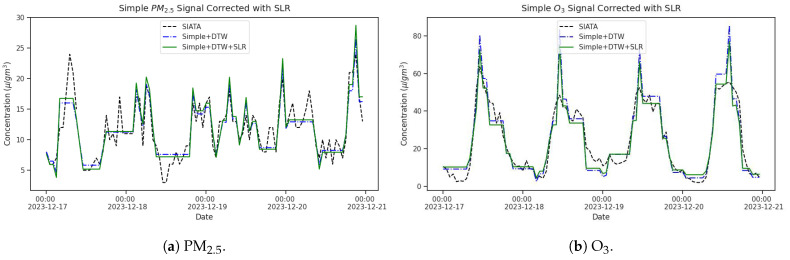
Corrected measurements for the Simple LCS signals compared to the reference values (SIATA). (**a**) PM_2.5_, (**b**) O_3_.

In this study, out of the various techniques for calibrating air quality sensors, we chose the SLR method to calculate linear coefficients and calibrate PM_2.5_ and O_3_ measurements performed during the intercomparison field campaign. Afterward, these calibration factors were applied to every unit in the in-situ 4DAir network. Figure 5 shows the calibrated PM_2.5_ and O_3_ signals for the Simple LCS installed alongside SIATA on 15 to 22 December 2023. Even though the calibrated and corrected signal lacks the exact temporal variability of reference signals, particularly for PM_2.5_ (Figure 5a), the compared signals now exhibit the same range of values and the correct daily cycle in both species.

Table 4 shows the linear coefficients for PM_2.5_ and O_3_ calculated using the SLR model, considering SIATA measurements during the intercomparison field campaign as a reference. As can be seen, there was no linearity between the LCS measurements and reference values if only raw data with preprocessing filters (raw column) or preprocessed signals corrected by temporal delay were used (DTW column), and the bias in both species was underestimated. However, using measurements corrected by the SLR method or corrected by combining the SLR and DTW methods, the linear coefficients correspond to a linear function of slope one and intercept zero, showing the best proportionality between the physical variable measured sensor output and the best corrections for offset error.

The results of the statistical analysis, before and after calibration, are shown in Table 5 for PM_2.5_ and O_3_ measurements during the intercomparison field campaign. Using the calibration procedure, the standard deviation (St. Dev.) reduces to 22% and 43% from raw values for PM_2.5_ and O_3_, respectively. Meanwhile, the Mean Absolute Error (MAE) and Root Mean Square Error (RMSE) improved up to 71% and 82% for both measurement sets. For calibrated signals of PM_2.5_ and O_3_, the MAE is 1.66 and 4.40 µg/m^3^, and the RMSE is 2.20 and 6.27 µg/m^3^, respectively. Similar works reported MAE ranged between 3.2 and 12 µg/m^3^, and RMSE from 4.1 to 15 µg/m^3^ for PM_2.5_; MAE ranged between 2.9 and 10 µg/m^3^, and RMSE from 6.0 to 26 µg/m^3^ for O_3_ [9,18,45,46,47,48,49,50,51]. Additionally, the correlation coefficients R^2^, Pearson R (R_p_) and Spearman R (R_s_) increased significantly, particularly for PM_2.5_ (from 0.01 to 0.79 for R^2^). Published works have reported values from 0.34 to 0.91 for R^2^ using multilinear regression for PM_2.5_ [6,11,45]. Therefore, the successful processing method for this work has been the combination of the SLR calibration and the temporal delay correction by the DTW analysis.

**Figure 6:**
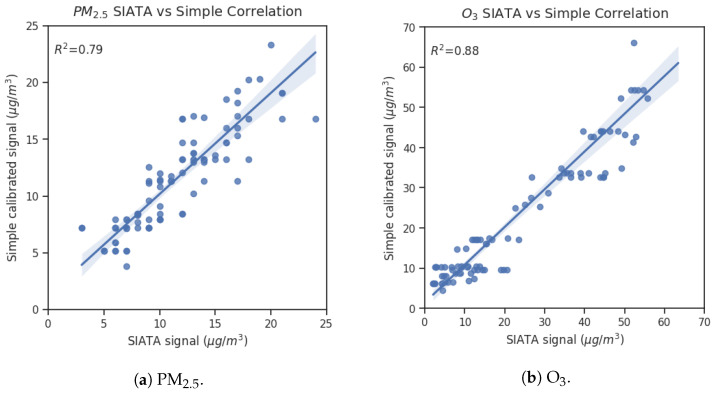
Scatter plots of the correlation between Simple unit data and reference data: (**a**) PM_2.5_ concentration comparison with reference sampler; (**b**) O_3_ concentration comparison with reference analyzer.

R^2^ enables evaluating how well the calibration improves the accuracy of the signals; however, it can be affected by outliers, unlike R_p_, which measures the linear relationships with relative changes, and R_s_ which uses absolute magnitudes for correlation calculation. This work selects the R^2^ as the evaluation metric since outliers are removed in pre-processing. Therefore, for PM_2.5_, this metric improved from 0.01 to 0.79, and for O_3_ changed from 0.72 to 0.88 through the evaluation and calibration process. Figure 6 shows the comparison between the Simple unit with the reference sampler for PM_2.5_ (Figure 6a) and with the reference analyzer for O_3_ (Figure 6b). It is found that the two datasets have a strong correlation.

In addition to the evaluation of PM_2.5_ and O_3_ in comparison with the reference instruments, basic meteorological variables (Temperature and Relative Humidity) were examined. Using the available SIATA values during the intercomparison field campaign, the 4DAir in-situ network meteorological data were calibrated with SLR results from the stations BLL and CPC since they were the closest active sites to the calibration site between 15 and 22 December 2023. The slope, intercept, and bias coefficients for temperature values are 0.6, 2.4, and −1.6, respectively. The corresponding values for relative humidity are 1.04, 23, and 0.0. Although several studies have shown that these meteorological variables affect electrochemical gas sensors [6,26] more than PM optical sensors [20], in this work, no correlation was found during the calibration field campaign due to the low temperature and relative humidity variability at the measurement site, 2.8 ºC and 15%, respectively. Furthermore, in Section 3, we reported the low variability during the measurement year (2.76 ºC and 11.4%, respectively).

### 3.2. 4DAir In-Situ Network Measurements

To set up the 4DAir in-situ network, nine Simple units were installed and operated between August 2023 and August 2024 in the stations shown in Figure 2, and they have been classified as rural (LNG and SAP), semi-urban (CPC, CLD and BLL), and urban (SDG, EAF, SJV, and POB) according to their location in the valley. A data archive was sent hourly from each unit to the database, with O_3_, PM_2.5_, and meteorological information. The linear coefficients obtained from SLR were used to calibrate the network data, assuming all units have equal performance.

Table 6 shows the percentage of data that were removed with the pre-processing filters described in Section 2.3. The percentage of eliminated data ranges from 3.1% to 9.8% across seven of the nine stations. This figure represents the simple average of the data removed for all measured variables in each station. For rural station LNG, a high 28% of data was removed; multiple blackouts during the year induced the sensor to save and send distorted data. In the case of the EAF urban station, 22% was removed from the data, as this was the only unit working on photoelectric energy, many power cut-offs would have reset the LCS, saving zero, negative, and low-confidence data.

A preliminary analysis was performed using filtered, corrected, and calibrated data from the 4DAir in-situ network, in order to determine accurate long-term values for PM_2.5_ and O_3_ concentrations in comparison with urban air quality reports; secondly, to examine the monthly evolution of pollutant species based on their location and, finally, to demonstrate the applicability of daily LCS measurements in locations that standard and governmental stations do not cover.

#### 3.2.1. Annual and Monthly Average Values from 4DAir In-Situ Measurements

Table 7 shows the yearly average values of PM_2.5_ and O_3_ concentrations, temperature, and relative humidity. These values are evaluated against data from nearby SIATA air quality stations where data are available. In Figure 2, the SIATA stations are identified in yellow pins as COP-CVID, CAL-JOAR, BEL-JEGA, and MED-PJIC; no SIATA stations are in rural areas. As previously mentioned, all SIATA stations are situated at the bottom of the valley, but the stations that are closest to the 4DAir semi-urban stations are COP-CVID, CAL-JOAR, and BEL-JEGA. Meanwhile, MED-PJIC is regarded as representative for comparison with urban stations.

For semi-urban and urban 4DAir stations, the arithmetic mean values of PM_2.5_ for the annual average are 19 µg/m^3^ and 20.5 µg/m^3^, respectively. These values are comparable to 15.2 µg/m^3^ and 18.1 µg/m^3^ reported by the local environmental authority [37,38,52]. Although the values reported in this paper are 25% and 13% higher than the standard information, urban stations show higher concentrations than semi-urban ones, as expected. Furthermore, the values of PM_2.5_ and O_3_ retrieved for rural stations were 3.47 and 9.40 µg/m^3^, respectively, representing these areas [37,53]. The maximum value of the POB station (23.25 µg/m^3^) for PM_2.5_ is explained by its proximity to multiple main roads and malls, its location surrounded by hills, and its poor ventilation. In addition, the higher values at the SJV station (24.77 µg/m^3^) correspond to the official air quality report (19.6 µg/m^3^), which shows that this station had the highest averages for daily and hourly cycles in 2023 [37].

Based on the mean O_3_ concentrations for semi-urban and urban 4DAir stations, we found that the yearly averages are 32.4 µg/m^3^ and 26.1 µg/m^3^, respectively. In this case, CAL-JOAR and BEL-JEGA stations are the only places to obtain official data [37,52]; the average value in 2023 was 28.6 µg/m^3^. Once more, the 4DAir in-situ network values are just 3.2% deviated from SIATA reports. This work also added information about O_3_ concentrations in semi-urban and rural stations, with an annual average of 32.4 and 26.1 µg/m^3^, respectively.

**Figure 7:**
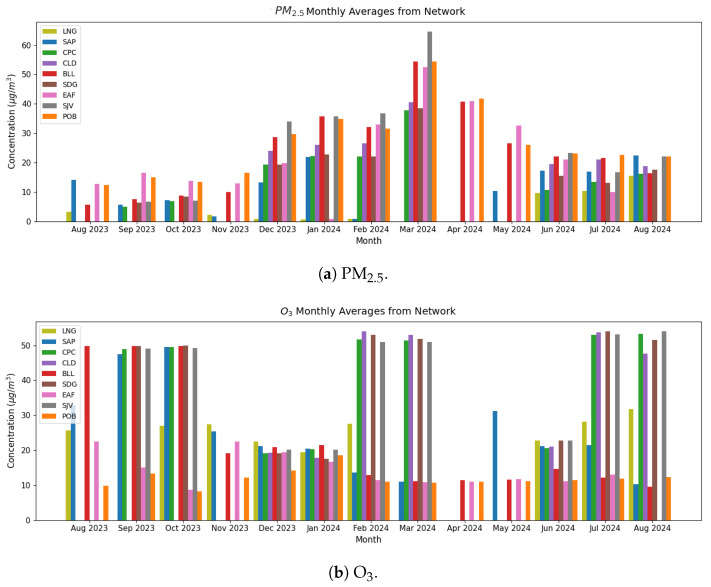
Monthly average concentration reported by the 4DAir network for PM_2.5_ and O_3_.

Figure 7 shows the monthly averages of PM_2.5_ (Figure 7a) and O_3_ (Figure 7b) concentrations for each 4DAir in-situ station data, where measurement durations vary from seven to twelve months according to installation time and data availability. We found that the PM_2.5_ concentration for semi-urban and urban 4DAir stations varied from small (6.2 and 14 µg/m^3^) and nearly constant (9.2 and 13 µg/m^3^) values between August and November 2023, to a monotonous increase from December 2023 (23 and 28 µg/m^3^) until reaching its peak in March 2024 (48 and 59 µg/m^3^). This data behavior is consistent with the government of the Aburrá Valley declaring an environmental contingency in March 2024 to control traffic flow. The April and May 2024 values (35 and 41 µg/m^3^) began to decline until June to August 2024, when they were nearly constant (19 and 22 µg/m^3^). Otherwise, SAP has a higher monthly average PM concentration throughout the year than LNG for rural stations, 22 and 9.6 µg/m^3^, respectively. The maximum monthly average was 64.64 µg/m^3^ at station SJV in March 2024. The SJV sector has also been identified as the maximum monthly average in 2023 by SIATA [37].

Regarding O_3_ concentrations in semi-urban stations CPC and CLD, we found a cyclic evolution, with maximum values in September and October 2023 (49 µg/m^3^ average), February to March 2024 (52 µg/m^3^ average), and July to August 2024 (52 µg/m^3^ average), with minimum values in November 2023 and January 2024 (19 µg/m^3^ average) and April to June 2024 (20.8 µg/m^3^ average). The BLL station showed less pronounced behavior due to a constant value of 49.9 µg/m^3^ average between August and October 2023; 20.5 µg/m^3^ average between November 2023 and January 2024, and 14.5 µg/m^3^ average between February and August 2024. Urban stations SJV and SDG showed the same cyclic variation for CPC and CLD stations. For EAF, POB, and rural stations LNG and SAP, the cycle was marginally discernible, with variations less than 20%; in the latter, LNG values exceeded SAP during the year under observation, to 35%. The lowest monthly concentration in October 2023 was in EAF and POB at 8 µg/m^3^. It is noteworthy that from December 2023 to January 2024, the average monthly O_3_ concentration for all rural, semi-urban, and urban stations is nearly constant at about 18 µg/m^3^. Because of the known cyclic relationship [54] and negative correlation [55] between O_3_ and NO_2_, in this study, from annual and monthly average values, we found that for CPC, CLD, SJV, and SDG stations, there could be less incidence of NO_2_ concentrations. In contrast, this polluted trace gas could be enhanced for SAP and LNG rural, as well as EAF and POB urban stations. Additionally, the period between December 2023 and January 2024 may have had the highest NO_2_ concentration throughout the valley during the measured year.

**Figure 8:**
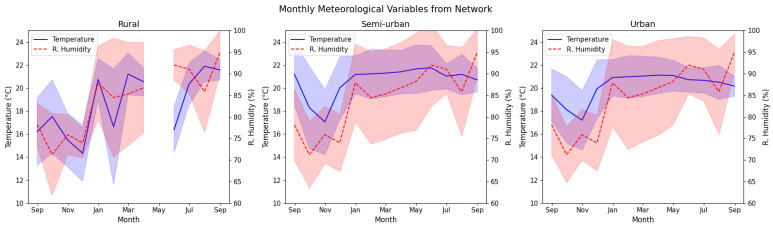
Monthly average of meteorological variables in the 4DAir network according to classified areas.

Finally, annual and monthly average values for meteorological variables (temperature and relative humidity) show stability as expected in a tropical climate (see Figure 8). The mean values of Temperature (and Relative humidity) in rural, semi-urban, and urban stations are 18.6 °C (84%), 20.6 °C (84.3%), and 20.1 °C (84.3%), respectively. Although rural stations had slightly lower temperatures during December 2023 and February 2024, around 12 °C, there are no significant differences between 4DAir stations. Regarding the relative humidity, the increase and stabilization from November 2023 is related to the rain increase due to the La Niña climate pattern. In this work, we found no considerable correlation between meteorological variables and PM_2.5_ or O_3_.

#### 3.2.2. Preliminary Analysis of Daily Average Values of PM_2.5_ and O_3_

**Figure 9:**
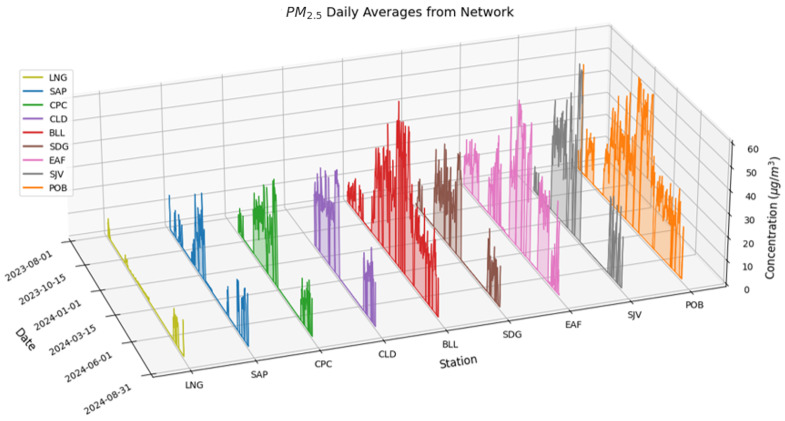
PM_2.5_ daily averages reported by the network.

Figure 9 shows the time series plot of 24h average values of PM_2.5_ for the in-situ 4DAir network between August 2023 and August 2024. The distribution range shows that PM_2.5_ concentrations rise from rural (8.6 µg/m^3^ average) to urban stations (25 µg/m^3^ average), and information loss is greater in rural stations due to power cuts. Remarkably, BLL between the semi-urban stations and SJV between urban stations have higher values. Since both stations are located on the western side of the valley, specific studies should be conducted to understand this pollution behavior in the Aburrá Valley.

Using the EPA 24-h standards [43] for PM_2.5_ and 8-h for O_3_, Table 8 shows the number of days each station surpassed the norm (#EXC), the maximum value, and the date at which the maximum was reached. We found that all monitoring stations (except for LNG) surpassed the norm for PM_2.5_ levels. Both CPC and CLD exceed the limit on 6.4% of the measured days, a proportion similar to that of SDG. In contrast, BLL, EAF, SJV, and POB exceeded the norm more frequently, surpassing it on approximately 18.5% of the measured days.

On the other hand, O_3_ maximum values did not exceed the threshold in any station; the date with the highest eight-hour average was August or September for all stations, except CLD, which was in March 2024. EAF, POB and LNG obtained a St. Dev. of 3–5 µg/m^3^; the first two are very close in location in a residential and commercial sector, while the latter is the highest above sea level and also in a rural area. The rest of the stations obtained a St. Dev. in a range of 15–18 µg/m^3^.

**Figure 10:**
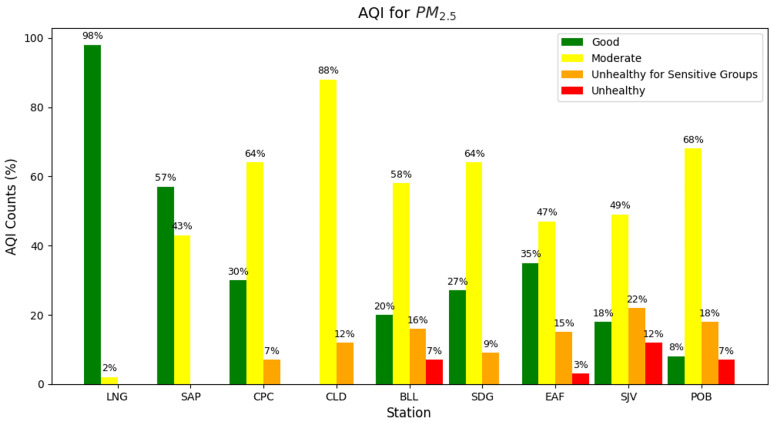
Air Quality Index (AQI) for PM_2.5_ from 4DAir network information.

Consequently, the AQI was computed for each station in the in-situ 4DAir network using the 8-h values for O_3_ and the 24-h values for PM_2.5_. The AQI for O_3_ was 100% green (Good) for all stations; thus, surface ozone concentrations do not represent any risk to the valley’s inhabitants according to these measurements. Nonetheless, Figure 10 shows that PM_2.5_ recorded AQI values ranged from moderate to unhealthy. Most of the values registered were in category yellow, except for LNG and SAP rural stations. On the other hand, SJV had the highest percentage of orange and red AQI, at 34%. According to official SIATA reports, the AQI result did not show red stations during the same period, though it did show up to 90% of yellow measures in most sectors and some with orange data. Spontaneous overestimations from the PM sensor due to saturation might cause these quantitative differences; nevertheless, more specific studies are necessary to understand the pollution dynamic inside the valley and its effects on people living in the hills.

## 4. Conclusions

A portable, easy-to-install device with a modular-based design to measure PM_2.5_ and O_3_ showed a viable way to acquire a large collection of air quality data at a significantly lower cost than current stationary sensors. This LCS named Simple Unit was developed as part of a study to examine urban air quality in a tropical valley with elevated areas, ranging from 1300 to 2800 m.a.s.l. Consequently, an LCS network called in-situ 4DAir was implemented. To evaluate the performance, the Simple Unit collected measurements colocated with standard instruments at the urban air monitoring station for one week in December 2023.

Data preprocessing was integrated into the validation stage, where filters were applied to remove outliers from raw measurements, i.e., zero values, negative values, and data outside the 95% confidence interval. Later, the calibration process consisted of a temporal delay correction and linear rescaling, referencing certified air quality sensor readings. The temporal delay was determined by the DTW function, which significantly improved the correlation between LCS and reference data. Afterwards, linear coefficients were calculated by the SLR method already used in [2], and applied to Simple data to compensate for any under or overestimation. For PM_2.5_, the R^2^ value increased from 0.1 to 0.8, while for O_3_, from 0.7 to 0.9. This result was satisfactory for the chosen calibration method since multilinear regression obtained R^2^ values for PM_2.5_ ranging from 0.34 to 0.91 in literature reports [6,9,11,45]. The MAE and RMSE for PM_2.5_ calibrated signals were 1.66 and 2.20, respectively; these errors are below the range found in the literature [9,18,45,46,47,48,49,50,51] and demonstrate the effectiveness of the process.

Moreover, the in-situ 4DAir network was installed to assess the reliability of sensors at various locations in the Aburrá Valley, from August 2023 to August 2024. The measurement sites were categorized as rural, semi-urban, and urban, with elevations ranging between 1400 and 2160 m.a.s.l. The analysis based on annual and monthly average values showed acceptable accuracy in studying PM_2.5_ and O_3_ concentrations, with deviations of 13% and 3.2%, respectively, compared to the nearest standardized measurement sites. Annual PM_2.5_ concentrations generally increased with decreasing elevation; however, SJV and POB urban stations showed the highest monthly and daily concentrations while not being at the lowest altitudes. Also, BLL (semi-urban) and POB (urban) stations frequently exceeded the maximum daily PM_2.5_ threshold of EPA, with 72 and 76 days, respectively, registered where this limit was surpassed. As for environmental conditions, temperature (20.2 ± 2.8 ºC) and relative humidity (84 ± 11.4%) exhibited minimal variation in this tropical valley, thus no quantifiable or observable correlation was found between these meteorological variables and PM_2.5_ or O_3_ concentrations in this study.

In summary, this work makes a significant contribution to addressing key challenges in developing LCS networks for air quality studies. It provides valuable insights into LCS performance under varying pollutant levels and the unique environmental conditions of mountainous and tropical regions. It also introduces a simple and efficient preprocessing and calibration method to ensure accurate data analysis. Through long-term monitoring at high altitudes above sea level and under tropical conditions of high temperature and relative humidity, the LCS Simple Unit demonstrated effective operation, stability, and reliable performance. This confirms its suitability for long-term air quality studies in tropical regions, enabling a better understanding of air quality variations and their impacts on health and biodiversity. Furthermore, this study addresses the critical gap in reliable air quality data for tropical regions and Latin America, where official monitoring stations are limited [22,23]. The methodological framework developed for validating LCS can be adapted to other geographic and environmental contexts, offering local governments a practical tool to implement cost-effective monitoring networks in both rural and urban areas.

One key challenge in this study was determining the temporal delay in the data and assessing its dependence on the type of low-cost sensor and standard measurement technology to evaluate the overall data quality. To address these issues, we recommend using the Dynamic Time Warping (DTW) method for data analysis, particularly in the context of field calibration processes involving optical particle counters. Furthermore, based on long-term measurements, we identified the need to develop a robust surveillance tool to detect and diagnose operational faults in network monitors.

Future work should focus on enhancing the capabilities of the Simple Unit sensors, particularly for measuring trace gases, to improve their performance under challenging environmental conditions. Expanding their capacity to monitor additional pollutants, such as NO_x_, SO_2_, CO, and volatile organic compounds (VOCs), would further increase their utility. Data from the 4DAir LCS network could also be incorporated into Chemical Transport Models to improve pollution forecasting through data assimilation. Finally, combining LCS data with remote sensing techniques could provide valuable insights into the impact of urban pollution on biodiversity, offering a more comprehensive understanding of environmental health.

## Figures and Tables

**Table 1:** Sensor Unit technical specifications.

Variable	Range	Type of Sensor	Reference	Brand
O_3_	0.02–10 ± 0.01 ppm	Electrochemical	110-4xx	Interlink Electronics
NO_x_	0.05–10 ± 0.01 ppm	MOS	MiCS-6814	SGX Sensortech
PM	0–999 ± 1 µg/m^3^	Laser dust	SEN0177	DFRobot
Temperature R. Humidity	−40–125 ± 3 °C 0–100 ± 2%	Digital	SHT3x-DIS	Sensirion

**Table 2:** Geographical information of 4DAir in-situ network stations.

Code	Location	Coordinates (Lat, Long)	Height (m.a.s.l.)	Urban/Rural
LNG	Llanogrande	6.1059065, −75.4571333	2090	Rural
SAP	San Antonio	6.1752556, −75.6677187	2160	Rural
CPC	Copacabana	6.347, −75.512	1400	Semi-urban
CLD	Caldas	6.0854164, −75.6325446	1765	Semi-urban
BLL	Bello	6.2372728, −75.5879966	1430	Semi-urban
SDG	Santo Domingo	6.287780, −75.544175	1700	Urban
EAF	EAFIT	6.2016808, −75.5783624	1475	Urban
SJV	San Javier	6.2621974, −75.6346136	1500	Urban
POB	El Poblado	6.1999692, −75.5549558	1475	Urban

**Table 3:** 4DAir in-situ monitoring network descriptive metadata.

Station	Installation Date	Duration (Days)	Days Measured
LNG	14 August 2023	359	40.74%
SAP	16 August 2023	357	49.16%
CPC	22 September 2023	320	47.98%
CLD	7 October 2023	305	54.58%
BLL	8 August 2023	365	75.66%
SDG	21 September 2023	321	46.13%
EAF	10 August 2023	363	76.46%
SJV	23 September 2023	319	45.11%
POB	10 August 2023	363	78.24%

**Table 4:** Linear coefficients before and after calibration.

Pollutant	Parameter	Raw	DTW	SLR	DTW + SLR
PM_2.5_ (µg/m^3^)	Slope	0.77	1.13	1.00	1.00
	Intercept	0.72	−1.42	0.00	0.00
	Bias	−0.04	−0.02	0.00	0.00
O_3_ (µg/m^3^)	Slope	0.57	0.87	1.00	1.00
	Intercept	−3.51	2.24	0.00	0.00
	Bias	−1.26	−3.99	0.00	0.00

**Table 5:** Statistical results before and after calibration of the simple unit in GRD station. Measurements were performed between 15 and 22 December 2023.

Pollutant	Parameter	Raw	DTW	SLR	DTW + SLR
PM_2.5_ (µg/m^3^)	St. Dev.	5.92	4.06	4.61	4.61
	MAE	5.99	1.61	4.78	1.66
	RMSE	7.47	2.13	6.13	2.20
	R^2^	0.01	0.79	0.01	0.79
	R_p_	0.12	0.89	0.12	0.89
	R_s_	0.07	0.91	0.07	0.91
O_3_ (µg/m^3^)	St. Dev.	31.71	20.55	17.96	17.96
	MAE	26.28	4.78	8.24	4.40
	RMSE	31.04	7.26	9.79	6.27
	R^2^	0.72	0.88	0.72	0.88
	R_p_	0.85	0.94	0.85	0.94
	R_s_	0.77	0.91	0.77	0.91

**Table 6:** Results of pre-processing filters applied by each measured variable in every 4DAir in-situ station.

Station	PM_2.5_ (%)	O_3_ (%)	Temperature (%)	R. Humidity (%)
LNG	35.33	4.63	23.56	50.21
SAP	26.78	0.64	2.09	9.49
CPC	4.40	2.90	2.68	4.98
CLD	6.43	3.56	3.01	3.53
BLL	10.07	0.23	7.95	8.80
SDG	4.96	3.23	2.61	3.69
EAF	23.60	3.90	28.70	32.98
SJV	5.44	3.70	7.61	8.77
POB	4.83	5.29	0.44	2.01

**Table 7:** Summary of annual mean values of hourly PM_2.5_ and O_3_ concentrations and meteorological variables in 4DAir in-situ network.

Station	PM_2.5_ (µg/m^3^)	O_3_ (µg/m^3^)	Temperature (°C)	R. Humidity (%)
LNG	3.47	25.85	16.4	87
SAP	9.40	26.28	19.9	80
CPC	16.01	38.20	22.0	81
CLD	21.05	35.92	20.5	88
BLL	19.97	22.96	21.3	80
SDG	16.64	37.66	20.0	79
EAF	17.15	15.16	20.5	82
SJV	24.77	38.44	20.7	80
POB	23.25	13.28	20.5	84

**Table 8:** Maximum daily values (MAX) in µg/m^3^ and number of surplusses (# EXC), according to The U.S. Environmental Protection Agency’s (EPA) standard; PM_2.5_ 24-h standard: 35 µg/m^3^, O_3_ 8-h standard: 137 µg/m^3^ [43].

Station	PM_2.5_ (µg/m^3^)			O_3_ (µg/m^3^)		
	# EXC	MAX	Date	# EXC	MAX	Date
LNG	0	15.5	6 August 2023	0	30.7	14 August 2024
SAP	2	37.4	22 January 2023	0	49.6	16 August 2023
CPC	17	45.2	5 March 2024	0	58.7	23 September 2023
CLD	23	45.7	7 March 2024	0	58.0	12 March 2024
BLL	72	71.0	7 March 2024	0	50.0	9 August 2023
SDG	18	45.6	7 March 2024	0	58.6	26 September 2023
EAF	58	63.4	29 February 2024	0	27.7	11 August 2023
SJV	56	75.2	8 February 2024	0	59.2	26 September 2023
POB	76	66.0	29 February 2024	0	22.5	11 August 2023

## Data Availability

The data that support the findings of this study are available from the corresponding author, Elena Montilla-Rosero, on reasonable request.
